# Supplementary material for: Multimodal analyses of immune cells during bone repair identify macrophages as a therapeutic target in musculoskeletal trauma
Source: Bone Res. 2024 Sep 29;12:56. doi: 10.1038/s41413-024-00347-3 (PMC11438896; doi:10.1038/s41413-024-00347-3)
Supplement: Supplementary file 1 — Supplemental material [file 41413_2024_347_MOESM1_ESM.pdf]

Multimodal analyses of immune cells during bone repair identify macrophages as a therapeutic target in musculoskeletal trauma

Supplementary material

Figure S1

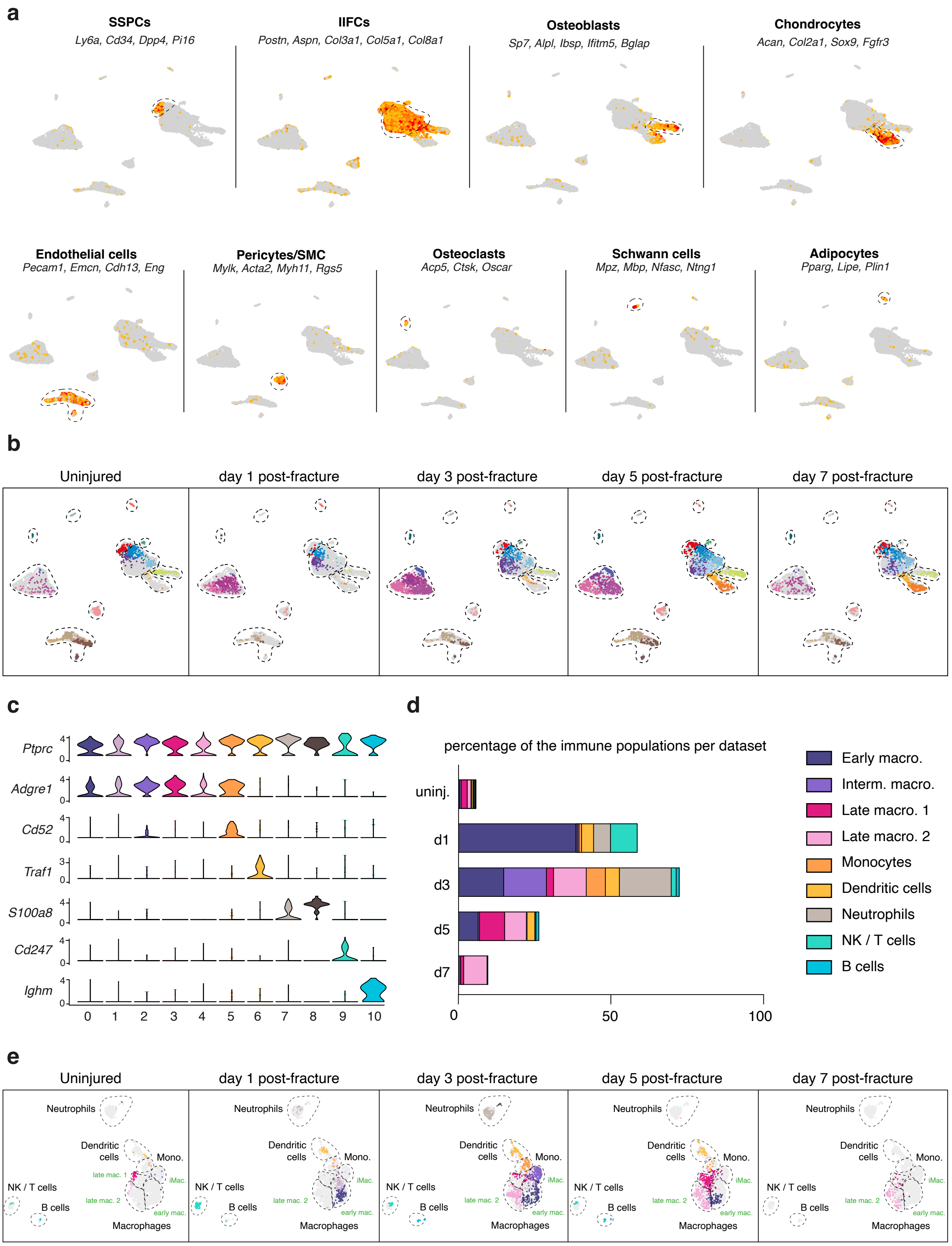

6

7 **Supplemental Figure 1: Immune cell atlas of bone regeneration**

a. Feature plots of the lineage score of the different cell populations in the combined fracture datasets. **b.** UMAP projection of the cells from the different time points in the integrated dataset. **c.** Violin plots of the expression of marker genes of the different immune cell populations. **d.** Percentage of cells in the different immune cell populations per time point. **e.** UMAP projection of the cells from the different time points in the integrated dataset.

Figure S2

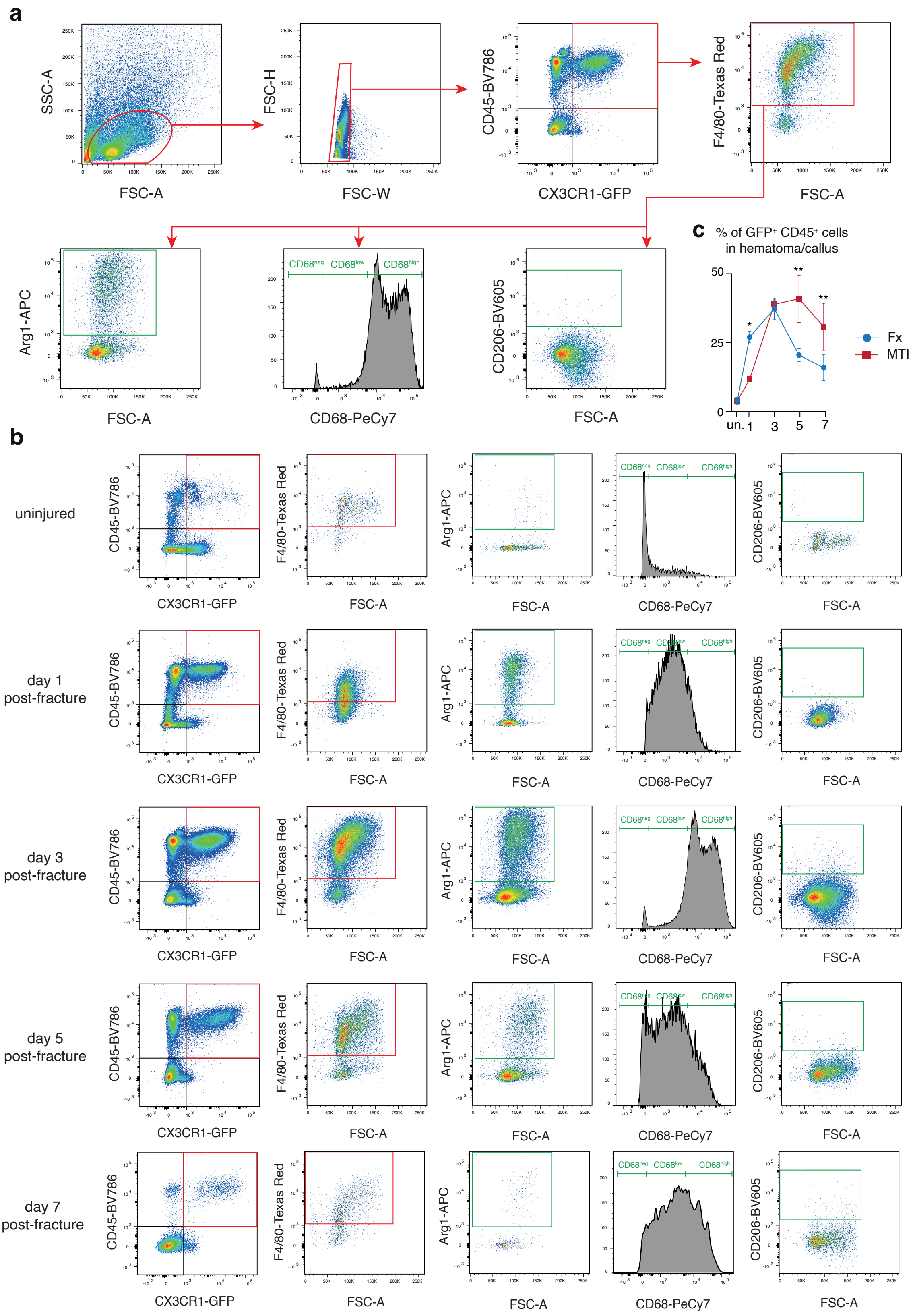

Supplemental Figure 2: Flow cytometry analyses of the macrophage dynamics after bone fracture

a. Gating strategy of the flow cytometry analyses of macrophages in the fracture environment. **b**. Flow cytometry analyses of macrophages in uninjured periosteum and muscle and in the fracture environment at days 1, 3, 5 and 7 post-fracture. **c**. Percentage of $CD45^{+}$ GFP^{+} cells in uninjured periosteum/muscle and in the fracture environment at 1-, 3-, 5- and 7-days post-fracture of *Cx3cr1*^{GFP} mice.

Figure S3

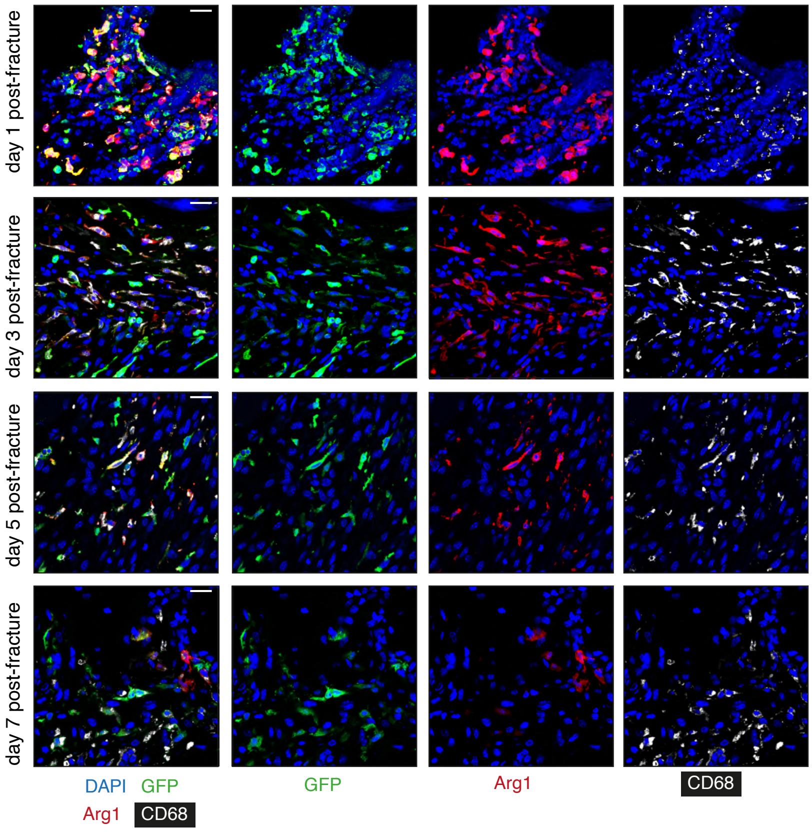

Supplemental Figure 3: Macrophages express both CD68 and Arg1 during the early steps of bone repair

Co-immunostaining of Arg1 and CD68 in hematoma and callus fibrosis at days 1, 3, 5 and 7 post-fracture of *Cx3cr1^GFP^ mice*. Scale bars: 25 µm

Figure S4

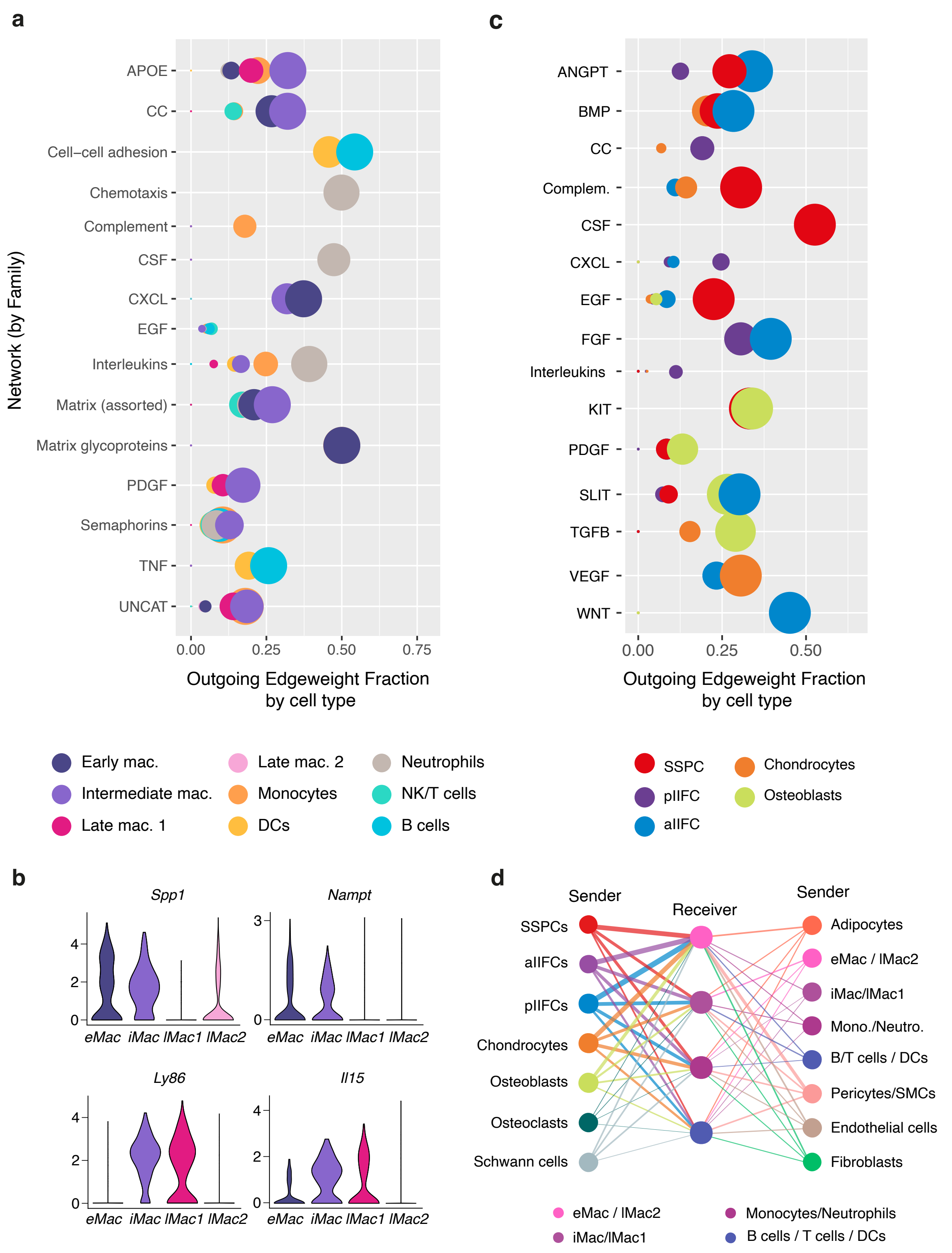

27

28 **Supplemental Figure 4: Paracrine interactions between immune cells and SSPCs during bone**

29 **repair**

a. Centrality analyses of the outgoing signals from the different immune cell populations after injury. **b.** Feature plots showing the expression of *Spp1* and *Nampt* by early macrophages (eMac) and intermediate macrophages (iMac) and the expression of *Ly86* and *Il15* by both iMac and late macrophages 1 (lMac1). **c.** Centrality analyses of the outgoing signals from the SSPCs, IIFCs, chondrocytes and osteoblasts after fracture. **d.** Interaction plot showing the interaction strengths from the different populations of the single nuclei dataset to the immune cells. The level of estimated interaction is displayed by the line thickness.

Figure S5

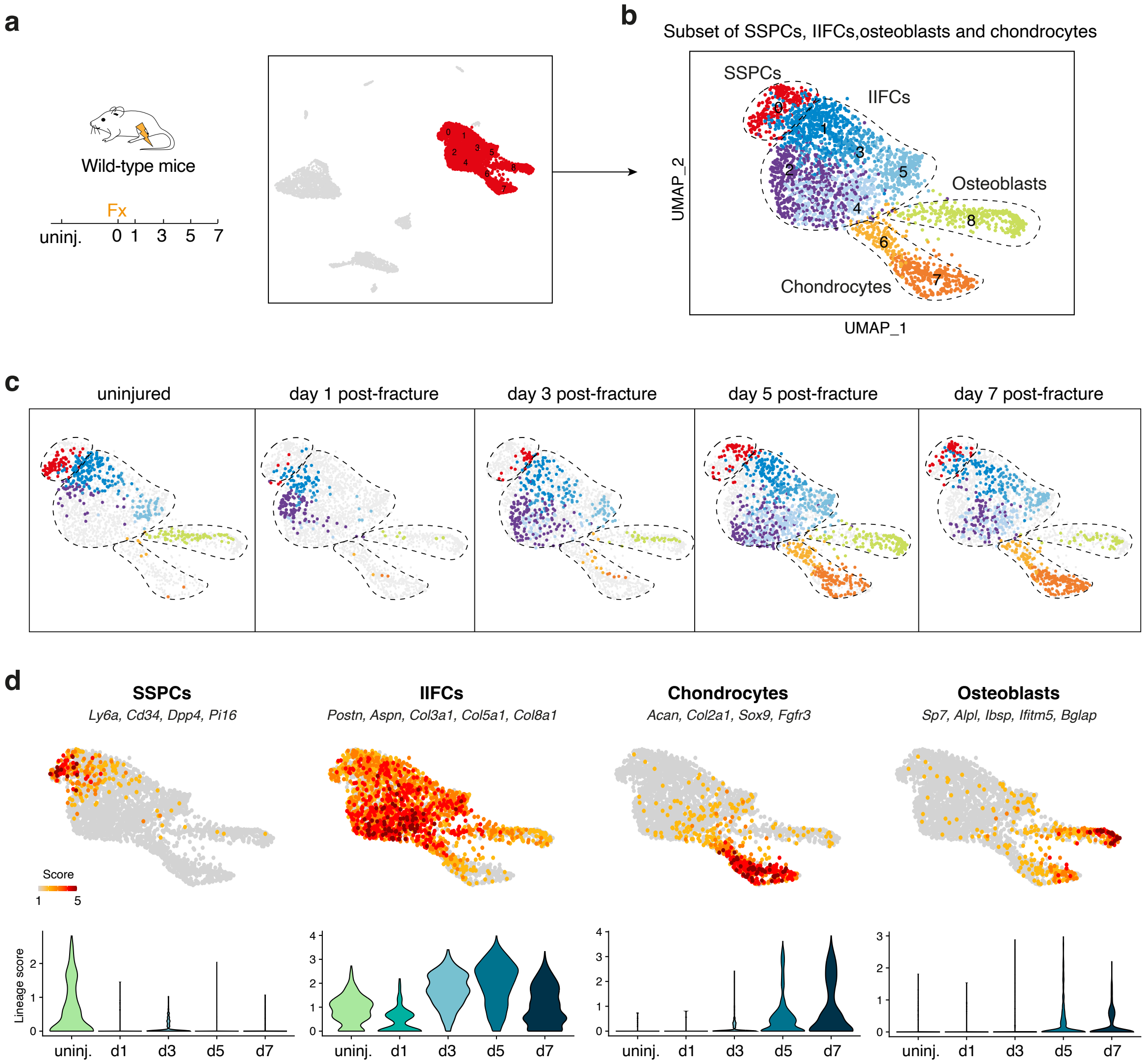

Supplemental Figure 5: Periosteal SSPCs differentiate via a common fibrogenic stage prior to osteogenesis or chondrogenesis

a. Experimental design. SSPCs, injury-induced fibrogenic cells (IIFCs), chondrocytes and osteoblasts from integrated uninjured, day 1, day 3, day 5 and day 7 post-fracture samples were extracted for a subset analysis. **b**. UMAP projection of color-coded clustering of the subset dataset. The four populations are delimited by black dashed lines. **c.** UMAP projection of the cells from the different time points in the subset of SSPCs, IIFCs, chondrocytes and osteoblasts. **d.** (top) Feature plots of the stem/progenitor, IIFC, chondrogenic and osteogenic lineage scores. (bottom) Violin plots of the lineage score per time point.

Figure S6

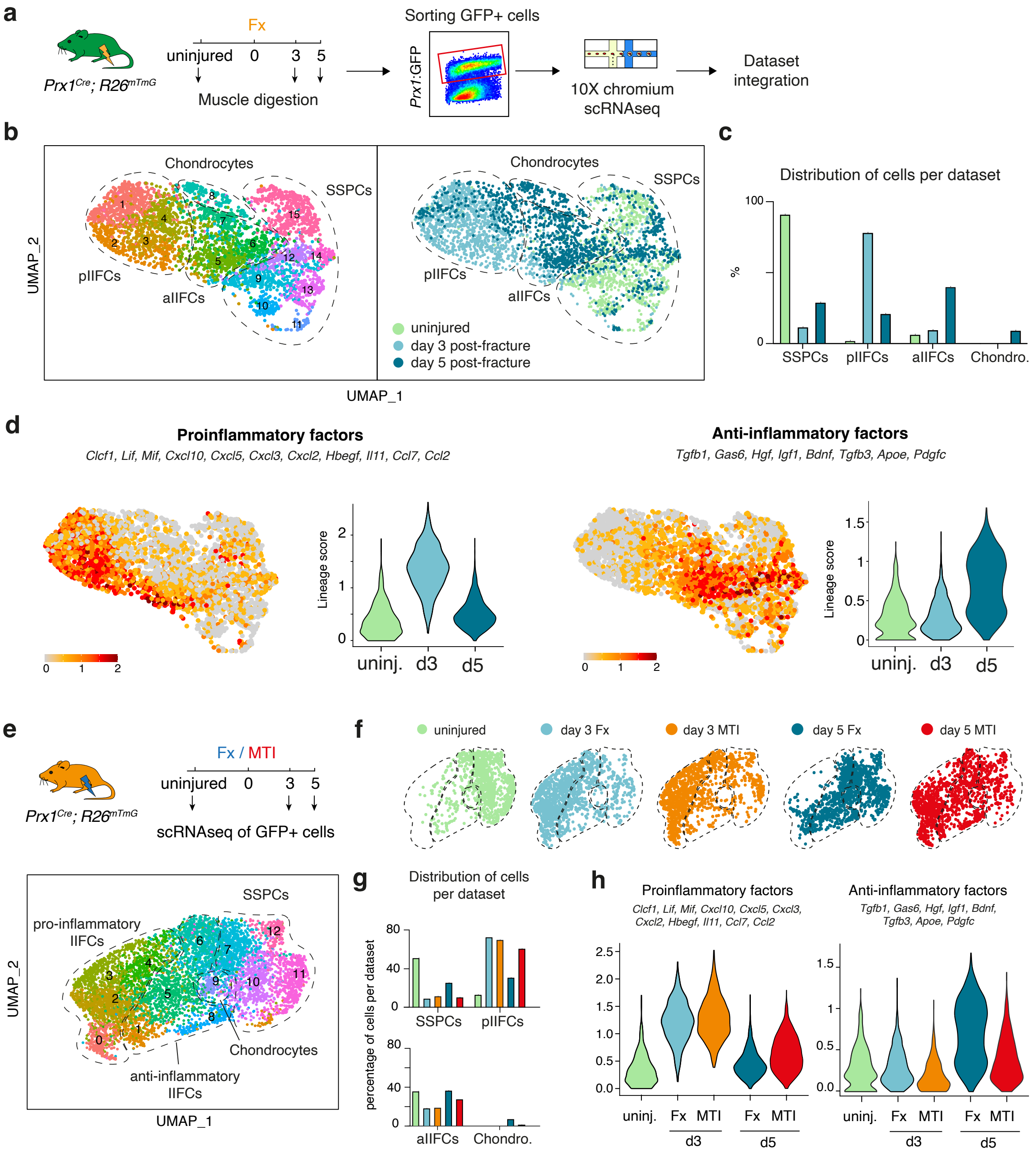

Supplemental Figure 6: The immune response of skeletal muscle SSPCs is altered after musculoskeletal trauma

a. Experimental design. Prx1-derived skeletal muscle cells were isolated from hindlimb skeletal muscles and sorted based on GFP-expression prior to scRNAseq[1]. **b.** Left, UMAP projection of color-coded clustering of Prx1-derived cells reveals 15 clusters defining 4 distinct populations (limited by a black dashed line). Right, UMAP projection of the integrated datasets generated from uninjured muscle and from muscles at day 3 and 5 post-fracture, colored by time point. **c.** Percentage of cells per population at each time point. **d.** Feature plots and violin plots of the expression of pro-inflammatory and anti-

inflammatory factors per time point. **e.** (top) Experimental design of scRNAseq experiment. Prx1-derived skeletal muscle cells were isolated from uninjured muscle and from muscles at day 3 and 5 post-fracture or MTI and sorted based on GFP-expression prior to scRNAseq [1]. (bottom) UMAP projection of color-coded clustering of Prx1-derived SSPCs and derivatives reveals 13 clusters defining 4 distinct populations (limited by a black dashed line). **f.** UMAP projection of the integrated scRNAseq dataset separated by dataset. **g.** Distribution of cells per population per time point. **h.** Violin plots of the expression of pro-inflammatory and anti-inflammatory factors per dataset.

Figure S7

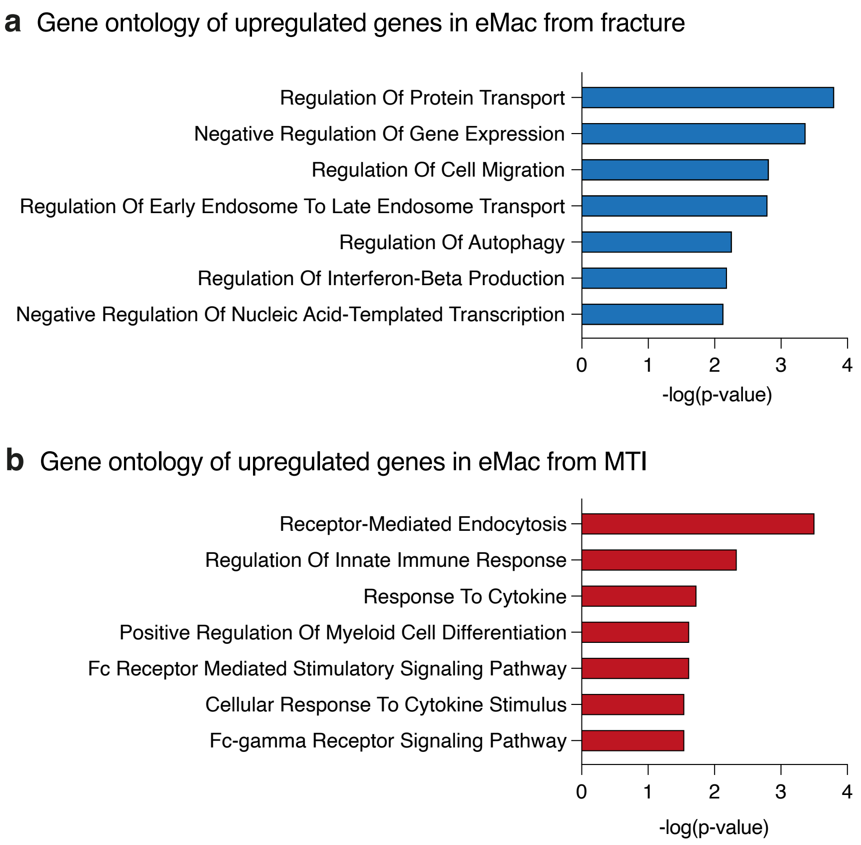

Supplemental Figure 7: Prolonged immune activity in early macrophages in MTI

Gene ontology analyses of upregulated genes in eMac at day 5 post-injury from the fracture dataset (**a**) and MTI dataset (**b**).

Figure S8

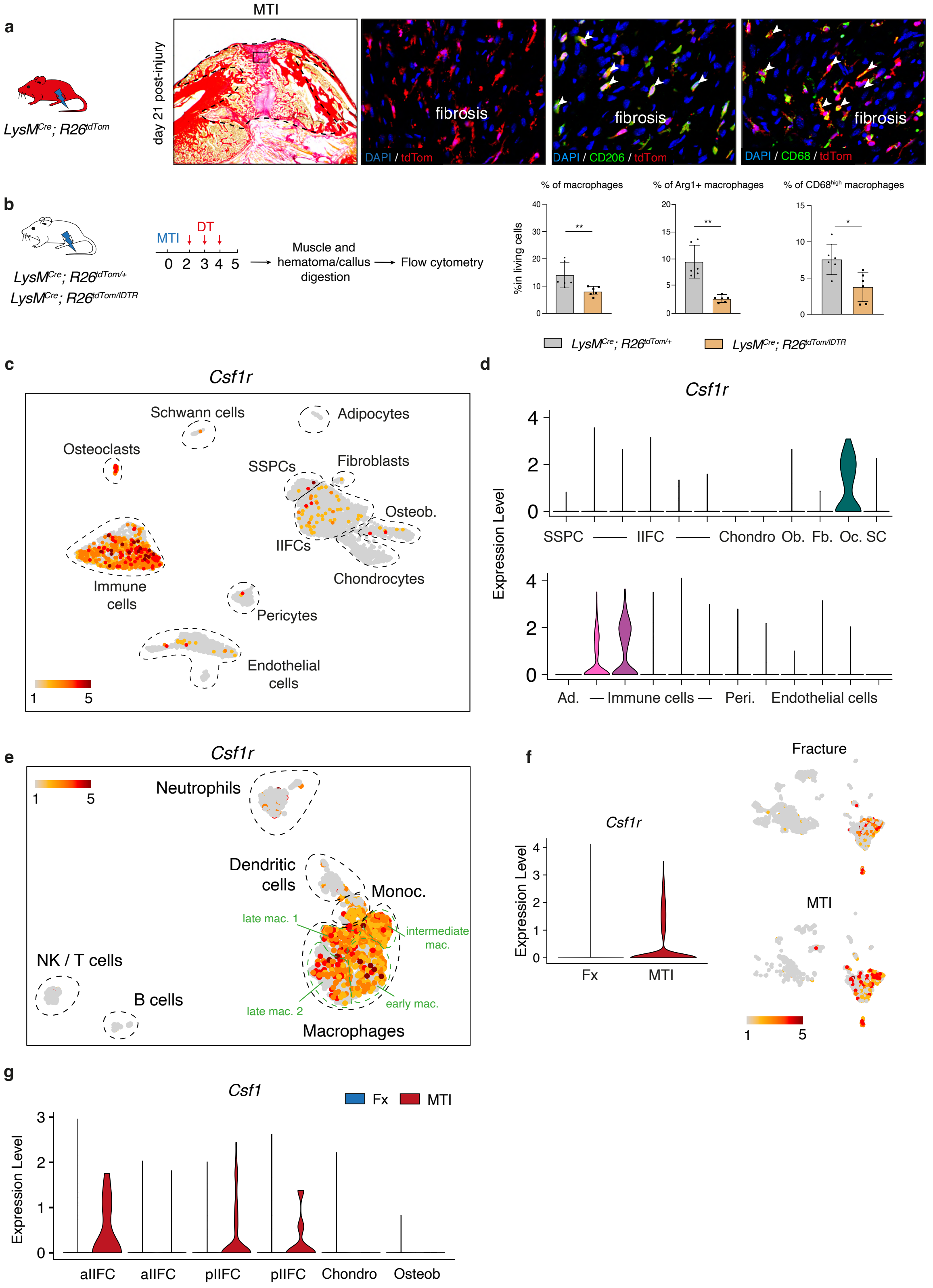

Supplemental Figure 8: Macrophages in MTI-induced fibrosis can be targeted in the *LysM*^{Cre} mouse model and express *Csf1r*

a. Callus section of 21 days post-MTI in *LysM*^{Cre}*; R26*^{tdTom} mice shows the presence of tdTom+ cells in the fibrotic tissue. Co-immunofluorescence of CD68 and CD206 shows that tdTom+ cells are CD68 and CD206 positive macrophages. **b.** Experimental design of macrophage depletion in *LysM*^{Cre}*; R26*$^{tdTom:/iDTR}$ mice and assessment of depletion efficiency by flow cytometry. Mice received DT injections at days 2, 3 and 4 post-MTI and cells were isolated from the fracture environment (hematoma/callus and muscle) at day 5. *LysM*^{Cre}*; R26*$^{tdTom/iDTR}$ mice showed a reduction in the percentage of macrophages ($CD45^+$ $Tomato^+$ F4/80+ cells) and the percentage of $Arg1^+$ and $CD68^{High}$ macrophages (n=6 per group). **c.** Feature plot of *Csf1r* expression in the complete integrated snRNAseq dataset from the periosteum and hematoma/callus at days 1, 3, 5, and 7 post-fracture. **d.** Violin plot of *Csf1r* expression in the different clusters from the integrated fracture dataset. **e.** Feature plot of *Csf1r* expression in the subset of immune cells. **f.** Violin plot and feature plots of integrated dataset separated by injury type of *Csf1r* expression in the day 5 post-fracture and post-MTI snRNAseq datasets. **g.** Feature plot of *Csf1* expression by IIFCs, chondrocytes and osteoblasts at day 5 post-fracture (blue) and MTI (red).

Antibody type	Use	Antigen	Reference
Primary	IF	rat monoclonal to mouse CD68	Biolegend, 137002
Primary	IF	rabbit polyclonal to mouse Arginase 1	Abcam, ab91279
Primary	IF	rabbit polyclonal to mouse CD206	Abcam, ab64693
Primary	IF	chicken polyclonal to GFP	Abcam, ab13970
Secondary	IF	Alexa Fluor 647 Goat anti-Rat IgG	Invitrogen, A21247
Secondary	IF	Alexa Fluor 555 Goat anti-Rabbit IgG	Invitrogen, A21428
Secondary	IF	Alexa Fluor 488 Goat-anti Chicken IgG	Invitrogen, A11039
Primary	FC	anti-CD45 BV786	BD Bioscience, 564225
Primary	FC	anti-F4/80	BD Bioscience, 565613
Primary	FC	anti-Arg1 APC	Invitrogen, 17-3697-82
Primary	FC	anti-CD206 BV605	Biolegend, 141723
Primary	FC	anti-CD68 Pe-Cy7	Invitrogen, 25-0681-82
Primary	FC	anti-F4/80	BD Bioscience, 567201

Supplementary Table 1: List of antibodies used for this study.

IF: immunofluorescence. FC: flow cytometry.

**Score**	**Genes**
SSPCs	*Ly6a, Cd34, Dpp4, Pi16*
IIFCs	*Postn, Aspn, Col3a1, Col5a1, Col8a1*
Osteoblasts	*Sp7, Alpl, Ibsp, Ifitm5, Bglap*
Chondrocytes	*Acan, Col2a1, Sox9, Fgfr3*
Immune cells	*Ptprc, Adgre1, Cd8, Cx3cr1*
Endothelial cells	*Pecam1, Emcn, Cdh13, Eng*
Pericytes/SMC	*Mylk, Acta2, Myh11, Rgs5*
Osteoclasts	*Acp5, Ctsk, Oscar*
Schwann cells	*Mpz, Mbp, Nfasc, Ntng1*
Adipocytes	*Pparg, Lipe, Plin1*
Macrophage pro-inflammatory secretome	*Cxcl2, Ccl7, Ccl2, Ccl9, Thbs1*
Macrophage pro-repair secretome	*Apoe, Hgf, Cxcl16, Tgfb1, Pf1, Pltp, Sema4d, Pros1*
Macrophage anti-inflammatory secretome	*Igf1, Gas6, Pdgfc*
Periosteal IIFC pro-inflammatory factors	*Cxcl2, Cxcl5, Ccl2, Ccl5, Ccl7, Clcf1, Csf1, Lif, Mif, Cit*
Periosteal IIFC anti-inflammatory factors	*Igf1, Bdnf, Tgfb3, Gas6, Hgf, Inhba*
Muscle IIFC pro-inflammatory factors	*Clc1, Lif, Mif, Cxcl10, Cxcl5, Cxcl3, Cxcl2, Hbegf, Il11, Ccl7, Ccl2*
Muscle IIFC anti-inflammatory factors	*Tgfb1, Gas6, Hgf, Igf1, Bdnf, Tgfb3, Apoe, Pdgfc*

Supplementary table 2: Lists of genes used for lineage/secretome score analyses.

References

1. Julien A, Kanagalingam A, Martínez-Sarrà E, et al. Direct contribution of skeletal muscle mesenchymal progenitors to bone repair. *Nat Commun*. 2021;12(1):2860. doi:10.1038/s41467-021-22842-5
